# Deposition of F-doped ZnO transparent thin films using ZnF_2_-doped ZnO target under different sputtering substrate temperatures

**DOI:** 10.1186/1556-276X-9-97

**Published:** 2014-02-26

**Authors:** Fang-Hsing Wang, Cheng-Fu Yang, Yen-Hsien Lee

**Affiliations:** 1 Department of Electrical Engineering, National Chung Hsing University, Taichung 40227, Taiwan; 2 Department of Chemical and Materials Engineering, National University of Kaohsiung, Kaohsiung 81148, Taiwan

**Keywords:** Deposition, FZO thin films, Secondary ion mass spectrometry

## Abstract

Highly transparent and conducting fluorine-doped ZnO (FZO) thin films were deposited onto glass substrates by radio-frequency (RF) magnetron sputtering, using 1.5 wt% zinc fluoride (ZnF_2_)-doped ZnO as sputtering target. Structural, electrical, and optical properties of the FZO thin films were investigated as a function of substrate temperature ranging from room temperature (RT) to 300°C. The cross-sectional scanning electron microscopy (SEM) observation and X-ray diffraction analyses showed that the FZO thin films were of polycrystalline nature with a preferential growth along (002) plane perpendicular to the surface of the glass substrate. Secondary ion mass spectrometry (SIMS) analyses of the FZO thin films showed that there was incorporation of F atoms in the FZO thin films, even if the substrate temperature was 300°C. Finally, the effect of substrate temperature on the transmittance ratio, optical energy gap, Hall mobility, carrier concentration, and resistivity of the FZO thin films was also investigated.

## Background

Transparent conducting oxide (TCO) thin films based on zinc oxide (ZnO) are promising for applications in various optoelectronic devices. In spite of extensive studies on preparation, characterization, and effect of doping on the properties of ZnO, certain effects of either some dopant or preparation procedures are still remaining unclear. However, ZnO-based thin films present a lot of advantages such as low material cost, non-toxicity, and high chemical stability under the hydrogen plasma as compared to tin-doped indium oxide (ITO) [1]. For that, transparent conducting ZnO thin films have already been extensively used in solar cells, light-emitting diodes, and liquid crystal displays as a substitute for ITO [2,3]. Much more interest has been given to TCOs based on ZnO such as undoped ZnO thin films [4], Al-doped ZnO (AZO) thin films [5], and Ga-doped ZnO (GZO) thin films [6] due to their stability under hydrogen plasma which makes them a potential candidate for solar cells' technology based on thin-film silicon. Fluorine, the ionic radius (0.136 nm) of which is similar to that of oxygen (0.132 nm), may be an adequate anion doping candidate due to lower lattice distortion compared with Al, Ga, and In, but comparatively few studies on fluorine-doped ZnO (F-doped ZnO) can be found in the past researches [7].

Many different physical and chemical deposition methods were used to investigate the properties of the F-doped ZnO thin films. For example, the F-doped ZnO thin films were deposited on Corning glass by radio-frequency (RF) magnetron sputtering of pure ZnO target in CF_4_-containing gas mixtures. The fluorine content in F-doped ZnO thin films increased with increasing CF_4_ concentration in sputter gas [8]. Treharne attempted to achieve substitutional doping of ZnO with F by using trifluoromethane (CHF_3_) as the partial pressures of Ar-H_2_-CHF_3_, where the constant ppH_2_ of 5% was used and the partial pressures of CHF_3_ were changed in the range of 0% to 7% [9]. The F-doped ZnO thin films could also be deposited on glass slide substrates using d.c. reactive magnetron sputter at room temperature, with the substrate holder at the floating potential using an Ar/O_2_(/F_2_) gas mixture by using the pure metallic Zn as target [10]. Anandhi et al. used the SnCl_2_ · 2H_2_O (0.1 M) and Zn(CH_3_COO)_2_ · 2H_2_O (0.2 M) as the host precursors and NH_4_F as the dopant precursor for the deposition of the F-doped SnO_2_ layer on the F-doped ZnO (FZO) layer to get the bi-layer thin films by using a simplified spray pyrolysis technique [11]. Rozati et al. used 0.4 M solution containing zinc acetate dehydrated as the host solution, which was dissolved in a mixture of double-distilled water, methanol (3:7 volume proportion), and acetic acid. They used the ammonium fluoride as the doped starting solution, with a fixed [F]/[Zn] ratio of 2 at.%; the F-doped ZnO thin films were also deposited by using a spray pyrolysis technique [12].

As we know, if the fluorine-based gases are used during the sputtering process, the environmental pollution problem and the problem of the chamber being etched are unavoidable. When the chemical deposition methods are used to deposit F-doped ZnO thin films on the substrate with large area, surface roughness and thickness uniformity are two important problems needed to overcome. In the past, Cao et al. prepared the highly transparent and conducting F-doped ZnO thin films on glass substrates by pulsed laser deposition using a sintered ZnO target containing 1 at.% zinc fluoride (ZnF_2_) as a function of oxygen pressure ranging from 0.01 to 0.5 Pa [13]. Ku et al. deposited the F-doped ZnO thin films at room temperature by using ZnO target with different ZnF_2_ contents. The fluorine content which increased almost linearly with increasing ZnF_2_ content in sputter target was expectable [14]. As physical vapor deposition method is used to deposit the TCO thin films, the substrate temperature will have large effect on their physical and electrical characteristics. Nevertheless, the two articles ([13,14]) do not investigate the effect of substrate temperature on the characteristics of the F-doped ZnO thin films.

Despite wide usage of magnetron sputtering technique in the fabrication of TCO thin films, only few studies have been reported on using the F-doped ZnO targets to deposit the FZO thin films by using sputtering technique. In this study, 1.5 wt% ZnF_2_ was added into ZnO powder to prepare the FZO target. The first important topic is that the FZO thin films were deposited by reactive RF magnetron sputtering on glass substrate by changing the substrate temperature. The effects of different substrate temperatures on the physical and electrical properties of the FZO thin films, including the crystallinity, surface and cross-sectional morphologies, carrier mobility, carrier concentration, resistivity, optical transmission spectrum, and optical band gap (*E*_g_) were all well investigated. Because the substrate temperature is increased from room temperature to 300°C, the second important topic is that the secondary ion mass spectrometry (SIMS) analysis is used to find the effect of substrate temperature on the fluorine content in the FZO thin films.

## Methods

**Figure 1:**
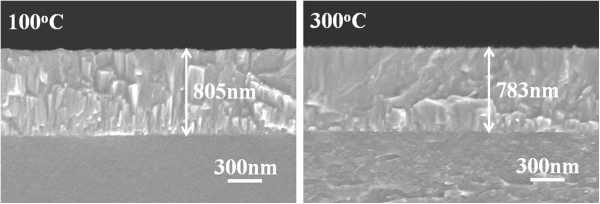
Cross-sectional observations of the FZO thin films as a function of substrate temperature.

The 1.5 wt% ZnF_2_ (99.995%) was mixed with zinc oxide powder (99.999%) to form the FZO composition. After being dried and ground, the FZO powder was calcined at 600°C for 1 h and then ground again and mixed with polyvinyl alcohol (PVA) as binder. The mixed powder was uniaxially pressed into pellets of 5-mm thickness and 54-mm diameter using a steel die. After debindering, the FZO pellet was sintered at 1,060°C for 3 h. The FZO thin films were deposited on 33 mm × 33 mm × 2 mm Corning 1737 glass substrates (Corning, NY, USA) using an RF sputtering system. Before the deposition process was started, the base chamber pressure was pumped to 5 × 10^−6^ Torr (detected by using MKS Baratron gauge, Andover, MA, USA), and the substrate temperature was changed from room temperature (RT) to 300°C. During the deposition process, the deposition pressure was controlled at 5 × 10^−3^ Torr, and only argon was introduced in the chamber; the flow rate of pure argon (99.999%) was approximately 20 sccm, and the deposition power was 150 W. The FZO thin films' thickness was measured using a Nano-View SEMF-10 ellipsometer (Ansan, South Korea) and confirmed by averaging five data obtained by field emission scanning electron microscopy (FESEM). The deposition rate was calculated from the measured thickness, while thin films' crystalline structure was identified by X-ray diffraction (XRD). By controlling the deposition time, a thickness of about 790 ± 30 nm was attained for all the FZO thin films, as Figure 1 shows. As calculated from the measured thickness, the deposition rate of the FZO thin films was linearly decreased from 2.37, 2.36, 2.35, to 2.34 Å/s as the substrate temperature was raised from RT, 100°C, 200°C, to 300°C, respectively. Those results suggest that substrate temperature has no apparent effect on the deposition rate of the FZO thin films. FESEM was also used to observe their surface and cross-sectional morphologies. The electrical resistivity was measured using a four-point probe, and the Hall effect coefficient was measured using a Bio-Rad Hall set-up (Hercules, CA, USA). The optical transmission spectrum was recorded by using a Hitachi U-3300 UV-vis spectrophotometer (Chiyoda, Tokyo, Japan) in the 300- to 800-nm wavelength range.

## Results and discussion

**Figure 2:**
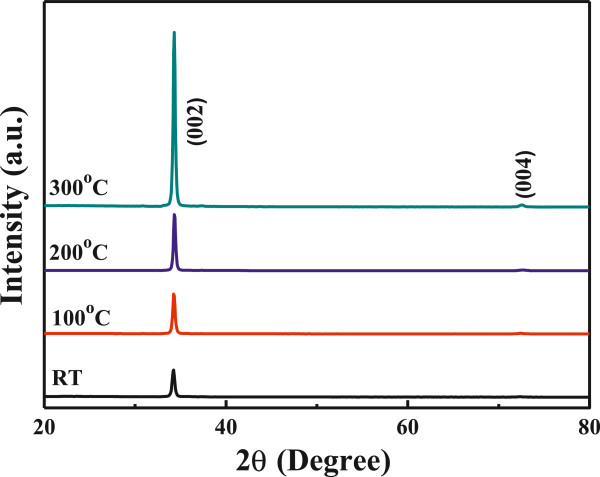
XRD patterns of the FZO thin films as a function of substrate temperature.

XRD patterns of the FZO thin films as a function of substrate temperature are shown in Figure 2. No extra phases involving fluorine compounds, such as ZnF_2_, were observed even for the RT-deposited FZO thin film. This can be attributed to low doping concentration of ZnF_2_. Even if all the FZO thin films exhibited the (002) peak, they really had different diffraction results. The diffraction intensity of (002) peak critically increased as the substrate temperature increased from RT to 300°C, and a weak (004) peak was also observed in the 300°C-deposited FZO thin films. Those results indicate that the *c*-axis of the FZO thin films is predominantly oriented parallel to the substrate normal. The images in Figure 1 show that the FZO thin films are very dense with wide-bar structure (for RT ~ 200°C-deposited FZO thin films) or nano-scale columnar structure (for 300°C-deposited FZO thin films) normal to the surface of the substrate, thus confirming the *c*-axis orientation growth as indicated by XRD patterns in Figure 2. The absence of additional peaks in the XRD patterns excludes the possibility of any extra phases and/or large-size precipitates in the FZO thin films. The (002) peaks of the FZO thin films prepared at substrate temperatures = RT, 100°C, 200°C, and 300°C were situated at 2*θ* = 34.24°, 34.24°, 34.31°, and 34.31°, respectively. The lattice constant *c* was calculated by using the 2*θ* value; as substrate temperatures were RT, 100°C, 200°C, and 300°C, the calculated lattice constants (*c*) were 0.5236, 0.5236, 0.5228, and 0.5228, respectively. As we know, the ionic radius (0.136 nm) of fluorine is similar to that of oxygen (0.132 nm). For that, as fluorine is used to substitute the sites of oxygen, the lattice constant *c* of the FZO thin films similar to that of the ZnO thin films is considerable.

As Figure 2 shows, as substrate temperatures were RT, 100°C, 200°C, and 300°C, the full width at half maximum (FWHM) values for the (002) peak of the FZO thin films were 0.320°, 0.294°, 0.280°, and 0.268°, respectively. The increase of relative diffraction intensity and the decrease of FWHM value of (002) peak with raising substrate temperature suggest that FZO thin films deposited at higher temperature have the better crystalline structure and the defects in the FZO thin films decrease with raising substrate temperature. This is because as higher substrate temperature is used to deposit the FZO thin films, the FZO particles or molecules can have larger active energy for the crystallization. For that, the number of thin films' defects decreases and the crystallization of the FZO thin films is improved; then, the FWHM value decreases.

**Figure 3:**
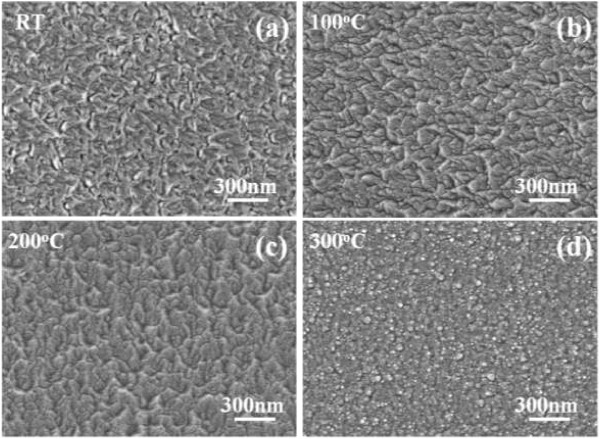
**Surface morphology of the FZO thin films as a function of substrate temperature. (a)** RT, **(b)** 100°C, **(c)** 200°C, and **(d)** 300°C.

Surface FESEM observations on the FZO thin films were taken to evaluate their surface morphology and lateral grain size, and the results are shown in Figure 3. As lower substrate temperatures were used, for example RT (Figure 3a), 100°C (Figure 3b), and 200°C (Figure 3c), the grain boundaries and roughness morphology were really observed on the surfaces of the FZO thin films. As higher substrate temperature was used, for example 300°C (Figure 3d), the grain boundaries were not easily observed and the densified and flat morphologies were really observed. As lower substrate temperature was used, the FZO particles or molecules have many nuclear points and have no enough active energy to aggregate together for thin films' flatness. As higher substrate temperature was used, the FZO particles or molecules will obtain the enough active energy for thin films' crystallization and flatness. The results in the XRD patterns match the variation in the morphology of the SEM observations that the different morphologies having in cross section (Figure 1) and the surface (Figure 3) of the FZO thin films will lead different crystallization results. The surface morphology in Figure 3d has a large difference as compared with those of Figures 3a,b,c. The cross-sectional observation in Figure 1 can be used to find the reason. The 100°C-deposited FZO thin films have a wide-bar structure, which will result in the cave-like surfaces. The 300°C-deposited ones have the nano-scale columnar aggregation structure, which will result in the small-grain surfaces.

**Figure 4:**
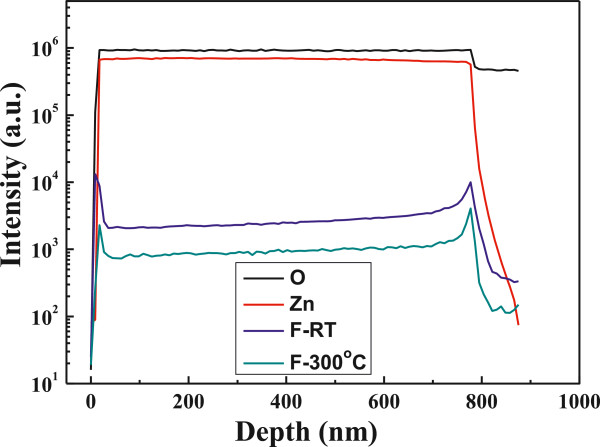
SIMS analysis of the depth profiles of Zn, O, and F elements.

Nakanishi et al. pointed out that as electron beam evaporation was used to deposit the ZnF_2_:Mn thin films, ZnF_2_ was oxidized to ZnO by residual O_2_ gas at a pressure of about 0.13 mPa [15]. In this study, ZnF_2_ was not likely to be transformed into ZnO because O_2_ gas was not introduced into the chamber during the sputtering process. SIMS is a high-sensitivity surface analysis technique for the determination of surface composition and contaminant analysis and for depth profile in the uppermost surface layers of a sample, and it can detect very low concentrations of dopants and impurities. Secondary ions formed during the sputtering process are extracted and analyzed using a mass spectrometer (usually a quadrupole or magnetic sector). For that, the SIMS analysis is used to find the distribution of Zn, O, and F elements, and the results are shown in Figure 4. Figure 4 proves that the thickness of the FZO thin films is around 790 nm (from the depth profiles of F and Zn). The concentrations of Zn and O elements are almost unchanged as the substrate temperature is raised from RT to 300°C. For that, only the concentrations of Zn and O elements of the RT-deposited FZO thin films are shown in Figure 4. Figure 4 also shows that fluorine can be detected in the 300°C-deposited FZO thin films, and the concentration of fluorine element of the RT-deposited FZO thin films is higher than that of 300°C-deposited FZO thin films. However, the relative fluorine concentration of the FZO thin films is lower than that of the prepared FZO composition. Because oxygen is not introduced during the deposition process, the vaporization of fluorine during the sintering and deposition processes is believed as the reason to cause this result.

When sputtering method is used to deposit the FZO thin films on a glass substrate, the FZO molecules are changed to plasma by the bombardment of Ar. Many defects are believed to be formed during the deposition process, which will inhibit electron movement. The crystallinity of the FZO thin films can be improved by many processes, the increase in the substrate temperature is believed the simplest process. However, as ZnF_2_ is added into ZnO as dopants and the FZO thin films are deposited at different substrate temperatures, three factors are believed to cause the variations of electrical properties of the FZO thin films. First, the higher substrate temperature provides more energy and thus enhances the mobility of deposition particles or molecules, which can improve crystallization and decrease the number of defects in the FZO thin films; the XRD pattern shown in Figure 2 has proven this result. Second, as the substrate temperature increases, the density of the FZO thin films increases and the barriers inhibiting electron transportation decrease; the SEM morphologies shown in Figure 3 has proven this result. Third, as we know, oxygen vacancies are formed during the deposition processes of the ZnO-based thin films, which will form the intrinsic n-type semiconductors. Too many oxygen vacancies may lead to an increase in the defect and scattering centers of the ZnO-based thin films, which will catch the electron and result in the decreases of carrier concentration and mobility. As the ZnF_2_ is added into ZnO as dopants, the fluoride will occupy the sites of ionic oxygen, and the problem in the decreases of carrier concentration and mobility can be improved. Figure 4 proves that even if FZO is deposited at 300°C, the fluoride can still be found in the FZO thin films.

**Figure 5:**
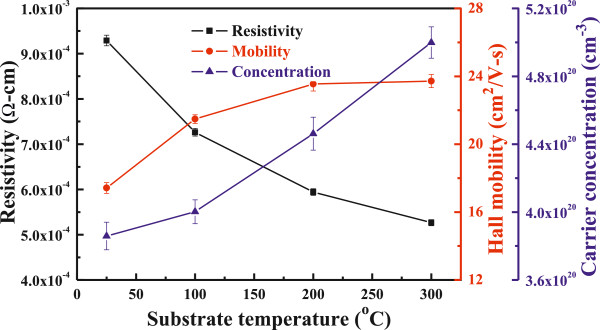
Hall mobility, carrier concentration, and resistivity of FZO thin films as a function of substrate temperature.

**Figure 6:**
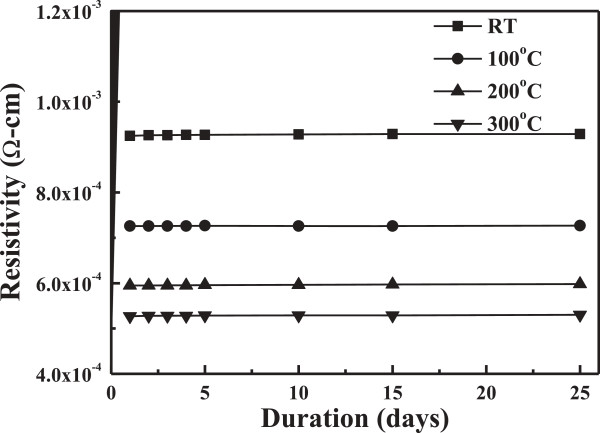
Variations of FZO thin films' resistivity as a function of duration time.

Both the carrier concentration (*n*) and the carrier mobility (*μ*) contribute to the conductivity (*ρ*), because *ρ* = 1/*neμ*. As Figure 5 shows for the FZO thin films deposited at different substrate temperatures, the carrier concentration and carrier mobility linearly increased with raising substrate temperature and reached a maximum concentration and carrier mobility of 5.00 × 10^20^ cm^−3^ and 23.72 cm^2^/V s at 300°C. A minimum resistivity of 5.27 × 10^−4^ Ω cm for the FZO thin films at a substrate temperature of 300°C is mainly caused by the carrier concentration and mobility being at their maximum. Figure 6 shows the variations of the FZO thin films' resistivity as a function of duration time. As Figure 6 shows, the FZO thin films have the stable resistivity values, because as the thin films are measured after duration of 25 days, the increase of resistivity is only 0.4%.

**Figure 7:**
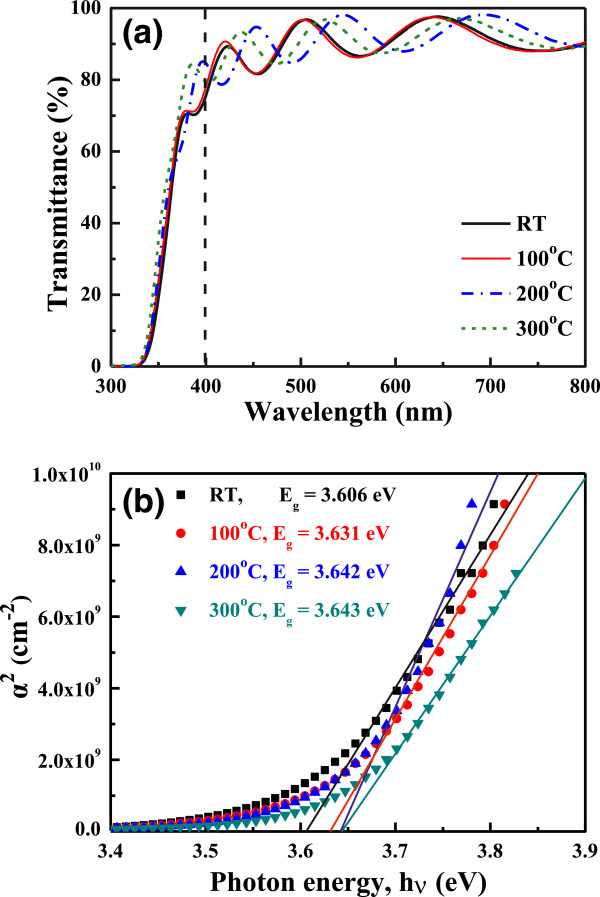
**Transmittance (a) and (*αhv*)**^***2***^**(where *a*** **=** ***αhv*) versus *hν-E***_**g **_**plots (b) of FZO thin films as a function of substrate temperature.**

Figure 7a shows the FZO thin films prepared at different substrate temperatures. The transmittance spectra are plotted against wavelengths in the region of 300 to 800 nm. As Figure 7a shows, the optical transmittance ratio at 400 ~ 700 nm is more than 90% for all thin films regardless of the variation of substrate temperature. The average transmittance ratio of the FZO thin films increased as the substrate temperature was raised. From the XRD patterns shown in Figure 2 and surface morphologies shown in Figure 3, the crystallization of the FZO thin films increases and the roughness of the FZO thin films is improved with raising substrate temperature. The two results prove that the improvements of the crystallinity and the densification are the reasons to cause the increase in the average transmittance ratio of the FZO thin films.

In the past, the determination of the optical band gap (*E*_g_) was often necessary to develop the electronic band structure of a thin-film material. However, using extrapolation methods, the *E*_g_ values of thin films can be determined from the absorption edge for direct interband transition. The absorption coefficient *α* was calculated using Lambert's law as follows:

(1) $$\alpha =\ln\left(1/T\right)/d$$

where *T* and *d* are the thin film's transmittance ratio and thickness. The absorption has a maximum at a high energy and decreases with optical energy in a manner similar to the absorption edge of semiconductors. Assuming that transition becomes constant at the absorption edge, the absorption coefficient *α* for simple parabolic scheme can be ascribed as a function of incident photon energy as [16]

(2) $$\mathrm{\alpha h\nu}\propto {\left(\mathrm{h\nu}-E_{g}\right)}^{n}$$

where *n* is a constant, *n* = 1/2 is the allowed direct transition and *n* = 2 is the allowed indirect transition, *hν* is the photon energy, and *E*_g_ is the optical band gap. Figure 7b shows the typical (*αhν*)^2^ versus *hν* plots of the FZO thin films as deposited at various substrate temperatures. The *n* value is around 1/2, and the linear dependence of (*αhv*)^2^ on *hν* indicates that the GZO thin films are direct transition type semiconductor. Figure 7b shows that as the substrate temperature increased from RT to 300°C, the *E*_g_ value increased from 3.606 to 3.643 eV.

**Figure 8:**
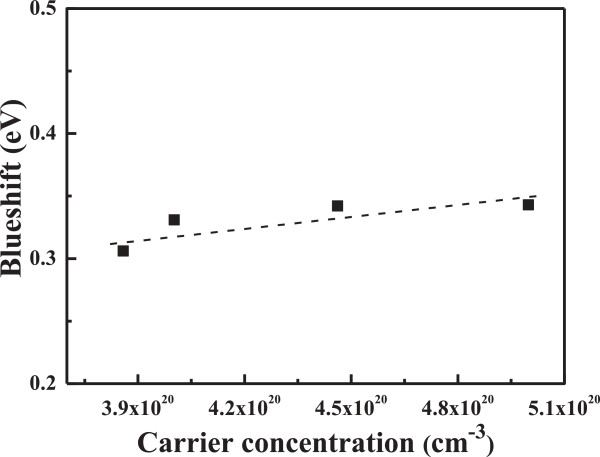
Relationship of the blue-shift and carrier concentration of the FZO thin films.

Figure 8 shows the relationship between the carrier concentration and *E*_g_ value, which presents that as the substrate temperature raises (or the carrier concentration increased), the absorption edge of the FZO thin films is blue-shifted (as calculated from 3.300 eV for ZnO thin films). This blue-shifting can be explained by the Burstein-Moss shift, a shift of the Fermi level into the conduction band, which enhances the optical band gap, as follows [17,18]:

(3) $$\Delta E_{g}^{\mathrm{BM}}=\frac{\hbar^{2}k_{F}^{2}}{2}\left(\frac{1}{m_{e}}+\frac{1}{m_{h}}\right)=\frac{\hbar^{2}k_{F}^{2}}{2m_{\mathrm{vc}}^{*}}$$

where *k*_F_ stands for the Fermi wave vector and is given by *k*_F_ = (3*π*^2^*n*_*e*_)^1/3^, *m*_*e*_ is the effective mass of electrons in the conduction band, and *m*_h_ is the effective mass of holes in the valence band, which can be simplified as $m_{\mathrm{vc}}^{*}$, the reduced effective mass. $\Delta \;E_{g}^{\mathrm{BM}}$ can be rewritten by *k*_F_ for the carrier concentration *n*_*e*_:

(4) $$\Delta E_{g}=\frac{h^{2}}{8m_{e}}{\left(\frac{3}{\pi }\right)}^{2/3}{n_{e}}^{2/3}$$

Equation 4 shows the important relationship between the degenerated semiconductor and the carrier concentration *n*_*e*_, where *m*_*e*_ is approximately equal to 0.28 *m*_0_, and *m*_0_ is the mass of the free electron [18]. In this study, the *n*_e_ value calculated from Equation 4 is around 0.692, which is close to the theoretical value of 0.667. When the wavelength is equal to 300 nm, the visible light absorbed by the thin films is due to a quantum phenomenon called band edge absorption. Burstein indicated that an increase of the Fermi level in the conduction band of a degenerated semiconductor leads to the energy band widening effect [17]. For that, the Burstein-Moss shift of the absorption edge to the shorter wavelength region is due to the increase in carrier concentration.

## Conclusions

FZO thin films had been successfully prepared by the RF magnetron sputtering under different substrate temperatures. A minimum resistivity of 5.27 × 10^−4^ Ω cm, with a maximum carrier concentration of 5.00 × 10^20^ cm^−3^ and a maximum Hall mobility of 23.72 cm^2^/V s, was obtained for the FZO thin films prepared at the substrate temperature of 300°C. The deposited FZO thin films had the stable resistivity values, because as the FZO thin films were measured after 25 days, only 0.4% increase in the thin films' resistivity was observed. The FZO thin films were uniform, and the average optical transmittance ratio in the entire visible wavelength region was higher than 90%, independent on the substrate temperature. As the substrate temperature increased from RT to 300°C, the *E*_g_ value increased from 3.606 to 3.643 eV, which indicated that the blue-shift effect really happened in the FZO thin films.

## Competing interests

The authors declare that they have no competing interests.

## Authors’ contributions

F-HW and Y-HL proposed an idea to deposit F-doped ZnO transparent thin films, helped in the deposition of the F-doped ZnO transparent thin films, participated in the experimental process, and helped in the data analysis. C-FY also proposed an idea to deposit F-doped ZnO transparent thin films, participated in the experimental process, helped in the data analysis, and wrote the paper. All authors read and approved the final manuscript.
